# Auditory temporal processing in healthy aging: a magnetoencephalographic study

**DOI:** 10.1186/1471-2202-10-34

**Published:** 2009-04-07

**Authors:** Peter Sörös, Inga K Teismann, Elisabeth Manemann, Bernd Lütkenhöner

**Affiliations:** 1 Department of Neurology, Münster University Hospital, Albert-Schweitzer-Str. 33, 48149 Münster, Germany; 2 Institute for Biomagnetism and Biosignalanalysis, University of Münster, Malmedyweg 15, 48149 Münster, Germany; 3 Department of Otolaryngology, Head and Neck Surgery, Münster University Hospital, Kardinal-von-Galen-Ring 10, 48149 Münster, Germany

## Abstract

**Background:**

Impaired speech perception is one of the major sequelae of aging. In addition to peripheral hearing loss, central deficits of auditory processing are supposed to contribute to the deterioration of speech perception in older individuals. To test the hypothesis that auditory temporal processing is compromised in aging, auditory evoked magnetic fields were recorded during stimulation with sequences of 4 rapidly recurring speech sounds in 28 healthy individuals aged 20 – 78 years.

**Results:**

The decrement of the N1m amplitude during rapid auditory stimulation was not significantly different between older and younger adults. The amplitudes of the middle-latency P1m wave and of the long-latency N1m, however, were significantly larger in older than in younger participants.

**Conclusion:**

The results of the present study do not provide evidence for the hypothesis that auditory temporal processing, as measured by the decrement (short-term habituation) of the major auditory evoked component, the N1m wave, is impaired in aging. The differences between these magnetoencephalographic findings and previously published behavioral data might be explained by differences in the experimental setting between the present study and previous behavioral studies, in terms of speech rate, attention, and masking noise. Significantly larger amplitudes of the P1m and N1m waves suggest that the cortical processing of individual sounds differs between younger and older individuals. This result adds to the growing evidence that brain functions, such as sensory processing, motor control and cognitive processing, can change during healthy aging, presumably due to experience-dependent neuroplastic mechanisms.

## Background

Auditory temporal processing, the precise detection of the temporal features of sounds, is a prerequisite for speech perception and reading (for a review, [1]). A deficit of auditory temporal processing has been suggested as a symptom in disorders as diverse as dyslexia [2] and autism [3]. Moreover, impaired speech perception is one of the major sequelae of aging [4]. Several lines of evidence suggest that not only peripheral high-frequency hearing loss, but also central deficits of auditory temporal processing contribute to the deterioration of speech perception in older individuals [5,6].

Numerous behavioral studies indicated an impairment of auditory temporal processing in healthy aging. Older listeners have more difficulties than younger listeners in detecting short gaps within speech and non-speech stimuli, a widely used behavioral technique for the assessment of auditory temporal resolution [7]. Age-related differences in gap detection appear to be independent of peripheral hearing loss because performance in gap detection is not correlated with audiometric thresholds [8,9]. Of particular importance for the perception of speech is that the discrimination of voice onset times can be impaired in the elderly [10]. The voice onset time refers to the time interval between the release of a consonant and the onset of vocal fold vibrations. Older listeners also show poorer performance in more complex behavioral tasks which evaluate the ability to discriminate changes in the timing of successive auditory stimuli [11,12]. Similar to gap detection, hearing loss does not significantly influence the performance in these tests [11,12]. There is evidence that these age-related deficits start relatively early in life. Deficits of temporal processing were already found in a group of 40–55 year-old individuals [13].

Considering these behavioral findings, we hypothesized that the cortical processing of rapidly recurring sounds, similar to the succession of syllables in speech, is impaired in the elderly. To test this hypothesis, we recorded auditory evoked magnetic fields (AEF) in healthy volunteers aged 20 to 78 years during short series of speech sounds using magnetoencephalography (MEG). MEG is an excellent tool for studying rapid temporal processing due to its high temporal resolution in the range of milliseconds. The recording of AEFs has a high reliability (test-retest reproducibility [14,15]) and a high validity (consistency with intracranial recordings [16,17]).

Data analysis focused on two major AEF components, the middle-latency P1m and the long-latency N1m, both originating from the superior temporal plane [16,18,19]. The N1m component is the strongest and most reliable component of the auditory evoked responses and is mainly influenced by temporal and physical aspects of a given stimulus [20]. We determined not only the latency and amplitude of the N1m and the location of the underlying cortical source, but examined also the decrement of the response amplitude that occurred when presenting series of four stimuli at short intervals, separated by a prolonged silent period [20]. The decrement of auditory evoked responses with rapid stimulation, also known as habituation [21,22], sensory gating [23,24], or attenuation, is believed to represent cortical filtering of irrelevant input [25]. Deficient encoding (or gating) of repeated stimuli might result in increased responses to repeated stimuli, as shown in patients with schizophrenia [26,27].

## Results

### N1m decrement

**Figure 1:**
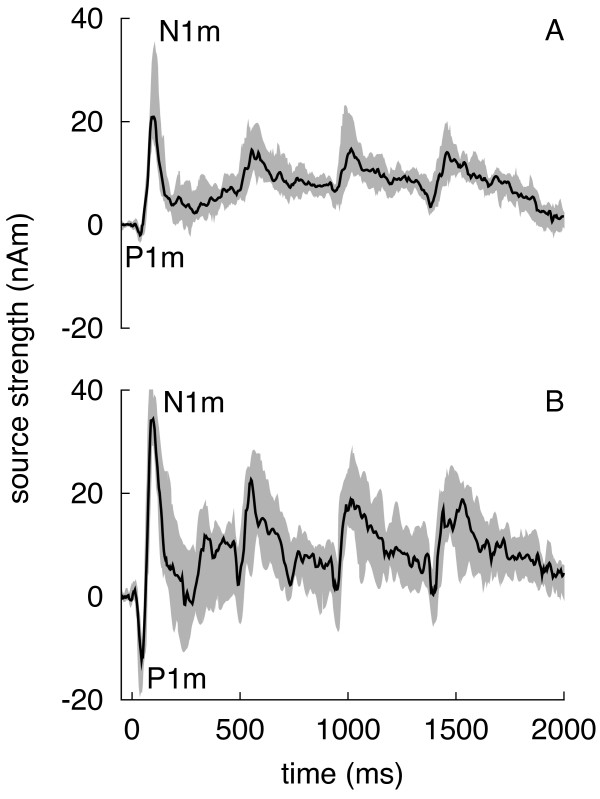
**Averaged AEF waveforms**. Averaged auditory evoked magnetic fields in 14 younger (A, age range: 20 – 27 years) and 9 older adults (B, age range: 60 – 78 years). The first P1m and N1m responses are marked. The black waveforms represent the median and the grey area the upper and lower 95% nonparametric bootstrapped confidence intervals.

Averaged MEG waveforms for a subgroup of younger adults (n = 14; mean age: 23 years; age range: 20 – 27 years) and a subgroup of older adults (n = 9; mean age: 66 years; age range: 60 – 78 years) are shown in Fig. 1. These waveforms represent the amplitude of the dipole moment for the source location of the first N1m peak over the entire epoch (-50 ms – 2000 ms). A prominent response, peaking about 100 ms after stimulus onset (N1m), and a preceding wave, peaking about 50 ms after stimulus onset (P1m), are visible in the averaged waveforms of both groups. The relative amplitude of the second N1m (the ratio between the amplitudes of the second and the first N1m response) was not significantly different between younger adults (median = 40%, range 0 – 77%) and older adults (median = 37%, range 20 – 51%; p = 0.69, Mann-Whitney test).

**Figure 2:**
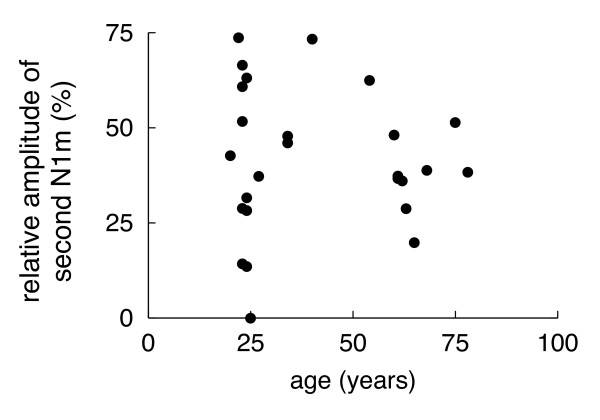
**Relative amplitude of the second N1m as a function of age**. No significant association between the N1m decrement (the relative amplitude of the second N1m to the first N1m) and age was found.

Fig. 2 depicts the relative amplitude of the second N1m response as a function of age in the entire sample (n = 28; mean age: 40 years; age range = 20 – 78 years). No significant correlation between the relative amplitude of the N1m and age was found.

### P1m amplitude

**Figure 3:**
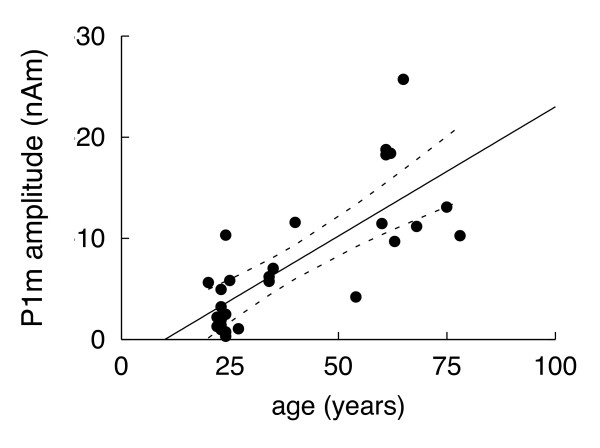
**P1m amplitude as a function of age**. P1m amplitudes are significantly larger in older than younger participants (linear regression, R^2^ = 0.56, F(1,26) = 32.78, p < 0.001). The black line represents the linear regression line and the dashed lines the confidence intervals.

Amplitudes of the first P1m are significantly larger in older (median = 13 nAm, range = 10 – 26 nAm) than in younger participants (median = 2 nAm, range = 0 – 10 nAm; p < 0.001, Mann-Whitney test). In the entire sample, the amplitudes of the first P1m were significantly correlated with age (ρ = -0.72, p < 0.001, Spearman's rank correlation; Fig. 3).

### N1m amplitude

**Figure 4:**
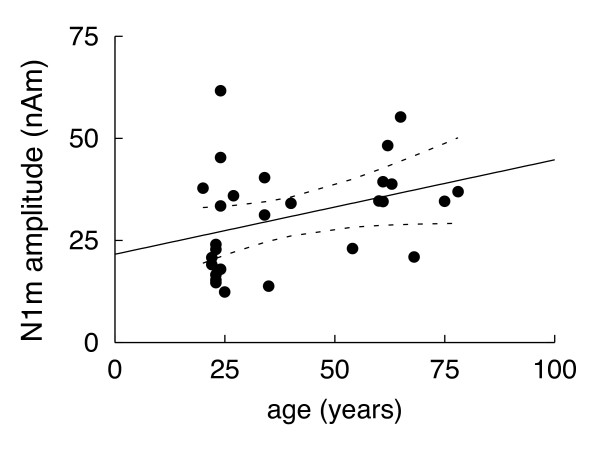
**N1m amplitude as a function of age**. N1m amplitudes tend to be larger in older individuals (linear regression, R^2^ = 0.13, F(1,26) = 3.77, p = 0.06). The black line represents the linear regression line and the dashed lines the confidence intervals.

Amplitudes of the first N1m are significantly larger in older (median = 37 nAm, range = 21 – 55 nAm) than in younger individuals (median = 22 nAm, range = 12 – 62 nAm; p = 0.03). In the entire sample, the amplitudes of the first N1m were significantly correlated with age (ρ = 0.42; p = 0.027; Spearman's rank correlation; Fig. 4).

### P1m and N1m latencies

The latencies of the first P1m and the first N1m were not significantly different between older and younger adults. The median P1m latency for older vs. younger individuals was 44 ms (range 37 – 57 ms) vs. 39 ms (range 30 – 57 ms; p = 0.22, Mann-Whitney test). The latency of the first N1m response was 94 ms (81 – 124 ms) in older and 101 ms (91 – 114 ms) in younger adults (p = 0.23, Mann-Whitney test). No significant correlations were found between age and the latencies of the first P1m and the first N1m when analyzing the entire sample (data not shown).

### Source locations

No significant correlation between age and source location and was found. Moreover, the source location was not significantly different between age groups.

### Hearing thresholds

**Figure 5:**
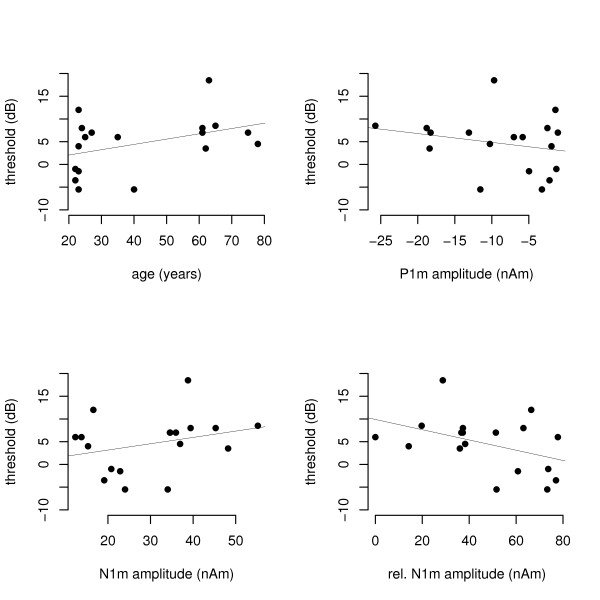
**Hearing thresholds**. A) Hearing thresholds for the vowel /a/ as a function of age in a relative dB scale. The line represents the linear regression line (R^2 ^= 0.16, F(1,16) = 3.04, p = 0.1). B) Hearing thresholds for the vowel /a/ as a function of the first P1m amplitude. Linear regression: R^2 ^= 0.05, F(1,16) = 0.94, p = 0.35. C) Hearing thresholds for the vowel /a/ as a function of the first N1m amplitude. Linear regression: R^2 ^= 0.08, F(1,16) = 1.44, p = 0.25. D) Hearing thresholds for the vowel /a/ as a function of the relative amplitude of the second N1m. Linear regression: R^2 ^= 0.18, F(1,16) = 3.42, p = 0.08.

Hearing thresholds for the speech stimulus used in the experiment were determined immediately before the MEG recordings on a relative dB scale. Hearing thresholds were 2.8 ± 5.8 (mean ± standard deviation) dB for the younger group and 8.1 ± 4.9 dB for the older group. Hearing thresholds tend to be higher in older individuals (Fig. 5A), but the effect is small (subjective hearing loss was an exclusion criterion). Participants with larger relative amplitude of the second N1m (i.e., with smaller decrement of the second N1m) tend to have lower hearing thresholds (Fig. 5D). No significant correlation was found between P1m and N1m amplitudes and hearing thresholds (Fig. 5B, 5C).

## Discussion

The results of the present study do not support the hypothesis that cortical processing of rapidly recurring speech sounds is impaired in healthy adults without subjective hearing impairment. The cortical processing of individual sounds, in contrast, differed between younger and older individuals and was characterized by significantly larger amplitudes of the middle latency P1m and the long latency N1m wave in older compared to younger participants.

### N1m decrement

The N1/N1m response is characterized by a pronounced amplitude decrease during repetitive stimulation [20]. When identical auditory stimuli are presented in pairs or short sequences (i.e., in a short-term habituation experiment), the second N1/N1m peak is considerably smaller than the first peak. Grand averages of electric [28,21,22] and magnetic recordings [29,15-34] demonstrate a decrease of the second N1/N1m amplitude to less than 50% of the amplitude of the first response. This amplitude decrease is frequently termed habituation [21,22] or sensory gating [23]. For the present report, however, we prefer the descriptive term "decrement" [15,32].

In the present study, the decrement of the N1m wave during a sequence of four vowel stimuli (ISI = 450 ms, sequence presentation rate = 0.2 Hz), was not significantly different between younger and older participants in a passive listening condition and in an environment with low acoustic noise. By contrast, two earlier ERP studies using pure tones of 50 ms duration [35] or harmonically enriched tones (75 ms duration) [36] provided evidence that the decrement of the P1-N1 and N1-P2 amplitudes [35] and the baseline-to-peak N1 amplitude [36] is smaller in older than younger adults. But there are important methodological differences. In the study performed by Papanicolaou et al. the participants attended to the stimuli [35], whereas in the present study they watched a self-selected silent movie that drew their attention and kept it on a relatively stable level. Selective attention has been shown to increase the amplitudes and the latencies of long-latency auditory evoked potentials [37-39]. In the study of Fabiani et al. [36], ERPs were recorded while the participants were reading a book, ignoring the stimuli. Although the study was similar to ours in this respect, the results are not easily comparable, because Fabiani et al. used the 200 ms time interval before the *first *stimulus in a sequence also as the baseline for the other stimuli in the sequence and did not correct for a baseline shift during the stimulus sequence [36].

As noted in the introduction, behavioral data indicate that the processing of rapid acoustic stimuli is impaired in the elderly [11,12,9,7,8]. The differences between our findings and previously published behavioral data might be explained, at least in part, by differences in the experimental settings. The experimental parameters used here differed from a natural listening situation. Although the magnetically shielded room containing the MEG system is not sound-proof, it attenuates external acoustic noise. Moreover, no competing sounds were presented during the acoustic stimulation. A major complaint of older individuals, however, is impaired speech perception in the presence of additional, distracting speakers. Numerous studies provided evidence that older listeners, even with relatively intact peripheral hearing, have more difficulties in understanding speech than younger adults when speech stimuli are masked by speech or noise [40,41].

The present study used a fixed onset-to-onset interstimulus interval (ISI) of 450 ms (presentation of 2.2 stimuli/s). This presentation rate was considerably lower than the rate at which individual sounds in normal speech are produced (5 – 10 syllables/s) [42]. In a pilot study, we used short (20 ms) sine tones and stimulated with an onset-to-onset ISI of 220 ms [43]. The short ISI resulted in an ill-defined baseline between evoked responses due to the superimposition of response waveforms, which made the quantification of P1m and N1m amplitudes unreliable. We cannot rule out the possibility that a faster presentation rate than used in the present study (i.e., > 2.2 Hz) might reveal significant differences between the N1m decrement in younger and older individuals. Repetition frequency is crucial because older listeners have more difficulties in accurately detecting words when the speech rate is faster than normal (for a review, see [44]).

### P1m and N1m amplitudes

The finding that P1m amplitudes are significantly larger in older than younger individuals corroborates earlier reports. Using AEPs, enhanced P1 components were detected in older adults [45-47,36]. Similarly, a more recent study used MEG to investigate auditory processing of elderly individuals and found larger P1m amplitudes [48].

Different mechanisms may contribute to increased P1/P1m amplitudes in older individuals. In aged mice with high-frequency hearing loss, the cortical representation of higher frequencies decreased while lower and middle frequencies became over-represented [49]. Thus, the loss of cochlear hair cells observed in human aging is probably accompanied by the reorganization of the auditory cortex resulting in a larger cortical representation of the frequency bands of speech [50], and therefore larger AEF amplitudes. A larger representation of speech sounds in aging is supposed to be associated with larger amplitudes of AEFs, representing the number of synchronously active neurons. In our study, however, a subjective hearing deficit was an exclusion criterion, and older participants had, on average, only slightly higher audiometric thresholds than younger participants. An alternative explanation for the larger P1m and N1m amplitudes in elderly humans is an impaired GABAergic neurotransmission [51,47], which would result in decreased subcortical and intracortical inhibition as demonstrated in aged rats [52].

We found significantly larger N1m amplitudes in older than in younger individuals (p = 0.03; Mann-Whitney U test). Moreover, the amplitudes of the first N1m were significantly correlated with age (ρ = 0.42; p = 0.027; Spearman's rank correlation). As the statistical analysis comprised multiple comparisons, these results have to be interpreted with caution, though. Previous studies reporting data on age-related changes of the N1/N1m amplitude were contradictory. Some investigators found no significant N1/N1m amplitude differences between younger and older adults [45,48], others found an enhanced N1m component [53]. Differences in the experimental setting (speech vs. pure tone stimuli of different frequencies, attentive vs. passive listening) complicate the comparison of these results.

### Methodological considerations

The estimation of P1m and N1m amplitudes might have been complicated by at least two factors, brain atrophy and increased hearing thresholds in aging. Brain atrophy is frequently found in older individuals [54,55] and may result in a deeper location of the P1m and N1m sources. Brain atrophy, therefore, might interfere with the quantification of source strengths because deeper sources tend to be estimated stronger than more superficial sources [56,57]. However, it is unlikely that brain atrophy introduced a considerable bias because no significant association between age and source location was found.

Another point to be considered is that all stimuli were presented at an intensity of 60 dB above the individual hearing threshold to achieve a comparable level of activation in the brain, basically independent of individual hearing thresholds. As a consequence, individuals with higher hearing thresholds received physically stronger stimuli than those with lower thresholds. While older individuals tended to have higher hearing thresholds than younger adults (p = 0.1; Mann-Whitney U test), we did not find a significant association between P1m/N1m amplitudes and hearing thresholds (Fig. 5), corroborating earlier ERP results [58]. For the present study we recruited healthy individuals without subjective hearing impairment, and we assured that their hearing threshold for the vowel stimulus was in the normal range, but we did not perform pure-tone audiometry. As a consequence, we cannot exclude that some of our participants might have had a moderate hearing loss at the frequencies corresponding to the higher formants in the speech signal. However, the effect of a possible high-frequency hearing loss on the results of our MEG recordings can be expected to be small, because the sound transmission between the speaker outside the magnetically shielded room and the ear piece inside the room attenuated higher frequencies anyway (see Methods).

Finally, recruiting healthy seniors with normal or near-to-normal hearing who qualified for an MEG experiment (no implanted metal or stimulation devices that may cause electromagnetic artefacts) was challenging for us. Thus, the sample size is relatively small (n = 14 in the group of younger, n = 9 in the group of older adults), and the conclusions based on the current study are limited by the small sample size and by the unequal age distribution of this sample. For future studies of auditory processing in healthy aging, a larger sample of participants is desirable, equally distributed over the adult life span. Such studies are expected to determine the age of onset and the further development of age-related changes in auditory processing.

## Conclusion

These results suggest that healthy aging is not necessarily associated with changes in the decrement of the major auditory evoked component, the N1m wave. The cortical processing of individual sounds, in contrast, was different between younger and older individuals, characterized by significantly larger amplitudes of the middle-latency P1m wave in older participants. All MEG recordings reported here were performed in a low-noise environment while participants watched a silent movie, although age-related central auditory disorders are often more pronounced in noisy situations. Thus, future studies on auditory temporal processing in aging should include auditory stimuli masked by noise [59,60].

## Methods

### Participants

Twenty-eight healthy, right-handed adults without subjective hearing impairment were included (15 men, 13 women, mean age = 40 years, age range = 20 – 78 years). All participants had normal audiometric thresholds for the auditory stimulus tested here. Participants gave their written informed consent to participate in the study. The study protocol was reviewed and approved by the Research Ethics Board, Medical Faculty, University of Münster, Germany. MEG recordings of some of the participants were published earlier as part of a healthy control group in a paper on auditory processing in stroke [32] and in a paper on the neurochemical basis of human cortical auditory processing [15].

### Auditory stimulation

The German vowel /a/ with a duration of 260 ms and a fundamental frequency of 234 Hz served as stimulus (unsmoothed, frequency of the first formant (F1) = 835 Hz, F2 = 1205 Hz). The stimulus was spoken by a female speech-language pathologist, recorded in a sound-proof chamber and stored on a computer. During the MEG recording, 160 sequences (or trains) comprising 4 repetitions of this stimulus were presented. The onset-to-onset interstimulus interval between the stimuli in a sequence was 450 ms. The onset-to-onset interval between sequences was pseudo-randomized between 4 s and 5 s. As the electromagnetic activation of the auditory cortex lasts for approximately 400 ms after the onset of a single transient stimulus, an interstimulus interval of 450 ms (or 2.2 stimuli/second) was chosen to avoid overlap between successive brain responses. The onset-to-onset interval between sequences (4.5 s) is long enough to allow a substantial recovery of the N1m component before the onset of the following sequence [22]. Immediately before each MEG measurement, the individual hearing thresholds were determined for the stimuli. A non-significant trend was observed for increased hearing thresholds in higher age (ρ = 0.43, p = 0.075, Spearman's rank correlation). The stimuli were delivered to a silicon earpiece in the right ear via speakers outside the shielded room and a plastic tube of 6.3 m length. Measurements with a 2 cm^3 ^ear-simulator (Model 4157, Brüel and Kjær, Nærum, Denmark) at the end of the plastic tubing indicated that the transmission of acoustic stimuli is relatively unimpaired up to a frequency of about 1500 Hz [61,62]. Spectra of narrow-band (1131 – 2262 Hz) and broad-band stimuli (400 – 6400 Hz) before and after passing through the sound delivery system (Fig. 3 in reference [62]) and the impulse response of the sound delivery system (Fig. 1 in reference [63]) were shown in earlier articles of our group. All stimuli were presented at an intensity of 60 dB above the individual hearing threshold.

### MEG data acquisition

Recordings were performed using a 37-channel biomagnetometer (Magnes I, BTi, San Diego, USA) in a magnetically shielded room and sampled at a rate of 512.4 Hz as published previously [15]. The participants were in a right lateral position with the body supported by a vacuum cushion to minimize head and body movements during the measurement. The sensor array was positioned over the left superior temporal cortex as closely as possible to the subject's head. Participants were instructed not to move their head, to stay awake, and to keep their eyes open. Participants listened passively to the stimuli to minimize the influence of inter-individual differences in attention and concentration. To ensure a stable passive listening condition, subjects watched a self-selected silent video which attracted their attention.

### MEG data analysis

After excluding epochs contaminated by artifacts, the magnetic waveforms were averaged and band-pass filtered (0.01–40 Hz). A single equivalent current dipole was calculated for each sampling point. To assess the cortical source of the N1m, dipole moments were averaged for a 30 ms time window around the N1m peak following the first stimulus in a sequence. This dipole was used to calculate the amplitude of the dipole moment over the entire epoch. For this calculation, the location and the direction of the N1m dipole were assumed to be constant (fixed-dipole approach). The N1m was identified as the strongest deflections in its typical latency range (70 – 150 ms). Based on the dipole moment over time, the peak amplitudes and peak latencies of the N1m responses were determined. In most participants, a baseline shift was detected from the beginning of the first response to the fourth response. A traditional baseline correction that uses a single time window of e.g. 200 ms before stimulus onset [61] would have resulted in confounded amplitude values. To ensure accurate quantification of amplitudes, dipole moments were therefore measured relative to a baseline that was defined as the mean value before the onset of each stimulus (-50 to 0 ms pre-stimulus) [15]. Baseline activity was not significantly different between age groups. In a subsequent step, the amplitude ratio of the second and first N1m (termed the relative amplitude of the second N1m) was calculated. To assess the variability of dipole moments, bootstrapped 95% confidence intervals were calculated over the entire epoch (Fig. 1).

### Statistics

Statistical testing was carried out for (1) the entire group of 28 participants and (2) two subgroups of younger (n = 14; mean age: 23 years; age range: 20 – 27 years) and older participants (n = 9; mean age: 66 years; age range: 60 – 78 years). Data are presented as median and range. Differences between group means were assessed using the two-tailed Mann-Whitney U test. To test for associations between variables, Spearman's rank correlation was calculated. Statistical analyses were performed with the statistical language R for Mac OS X .

## Authors' contributions

PS, IKT, EM, and BL designed the study, interpreted the results, and drafted the manuscript. PS, IKT, and EM recorded and analyzed the MEG data. All authors read and approved the final manuscript.
